# Bloody diarrhea, STEC infection, and HUS in the molecular microbiology era

**DOI:** 10.1007/s00467-025-06930-y

**Published:** 2025-08-23

**Authors:** Letizia Dato, Maria Cristina Mancuso, Laura Daprai, Thomas Ria, Daniele Rossetti, Annapaola Callegaro, Gianluigi Ardissino

**Affiliations:** 1 Division of Pediatrics, Department of Health Sciences, Università del Piemonte Orientale, Novara, Italy; 2 Microbiology and Virology Unit, Fondazione IRCCS Ca’Granda Ospedale Maggiore Policlinico, Milan, Italy; 3 Center for HUS Prevention, Control and Management, Fondazione IRCCS Ca’ Granda Ospedale Maggiore Policlinico, Milano, Italy

**Keywords:** Bloody diarrhea, STEC-HUS, Shiga toxin, Molecular diagnostic, Thrombotic microangiopathy

## Abstract

Hemolytic uremic syndrome (HUS) associated with Shiga toxin-producing *Escherichia coli* (STEC) infection remains a major individual and public health challenge throughout the world causing substantial personal, social, and economic burdens. In Western countries, bloody diarrhea (BD) in children is related to STEC infection in at least 6% of cases (rising to 15–20% in summer). This infection may turn into STEC-HUS in about 15% of patients. The widespread use of molecular microbiology leads to the diagnosis of STEC infection before the onset of HUS in an increasing number of patients. The anticipation of the diagnosis creates a window of preventive and/or therapeutic opportunities that include rehydration of dehydrated patients and/or volume expansion that have both proven to mitigate the severity of HUS. Traditionally, antibiotics are not recommended in STEC infections, but recent data suggest a promising potential preventive role for bacteriostatic agents (e.g., azithromycin), if they are given early in the course of the infection. It is recommended to test all children with BD for Shiga toxin (Stx) encoding genes, actively infuse Stx-positive patients with isotonic crystalloid solutions and carefully monitor them with urine dipstick for hemoglobinuria to early identify those who might eventually develop HUS. The suggested approach will increase the detection of STEC-infected patients thus enhancing our knowledge of the mechanisms of disease spreading. The early diagnosis of STEC infection combined with the mentioned therapeutic opportunities will hopefully decrease the number of children suffering from HUS, its case fatality rate and/or improve its short- and long-term outcomes.

**Graphical abstract:**

**Figure:**
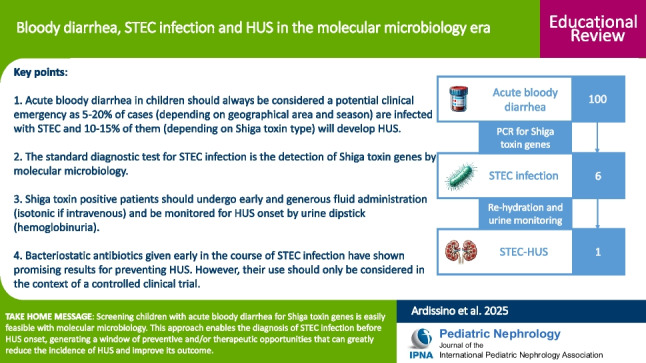
A higher resolution version of the Graphical abstract is available as Supplementary information

**Supplementary Information:**

The online version contains supplementary material available at 10.1007/s00467-025-06930-y.

## Introduction

Despite significant advances in scientific knowledge and medical technology over recent decades, hemolytic uremic syndrome (HUS) associated with Shiga toxin-producing *Escherichia coli* (STEC) infection (STEC-HUS) remains a major individual and public health challenge worldwide, presenting substantial personal, social, and economic burdens.

This paper aims to summarize current knowledge on STEC-HUS, with a particular focus on the early stages of the disease, prior to the onset of TMA, as this phase represents a crucial window for treatment and prevention. Advances in molecular microbiology have made early detection of STEC widely accessible. If employed effectively, early testing can help to identify potential sources of infection within the population and allow early risk stratification for STEC-HUS. This proactive approach enables timely implementation of interventions that can mitigate the severity of HUS or even prevent its development.

## Bloody diarrhea and STEC infection

In children, STEC infection often presents with a distinctive symptom that quickly draws parents’ attention: blood in the stool. Acute bloody diarrhea (BD) is defined as the onset of diarrhea within the previous 10 days, accompanied by visible blood in at least one bowel movement, either observed by a healthcare professional or reported by caregivers [1]. Approximately 80–90% of STEC-HUS cases are associated with acute BD. However, cases arising from non-hemorrhagic diarrhea tend to be less severe, generally leading to better outcomes [2, 3]. Given this, the current paper focuses on the diagnostic process for the majority of cases which present with acute BD.

In Western countries, the incidence of simple diarrhea in children is approximately 600–900 events per 1000 children per year, while BD affects no more than 3.5 per 1000 children annually [1, 4]. Molecular microbiology has revealed that BD has a well-defined bacterial etiology in approximately 60% of cases [5]. Epidemiological data justify Philip Tarr’s statement that BD in children should always be considered a potential clinical emergency [6]. In Europe, this assertion is based on the observation that STEC accounts for 5–6% of BD cases, increasing to 15–20% during late summer–early fall [7]. Data from our center, based on thousands of tested BDs, show that 7.1% are due to STEC infection (Table 1). A similar investigation performed in Utah on a limited number of cases of BD (*n* = 111) during a limited time period (18 months) reported a relative percentage of 13% of STEC infection [5], while a prospective multicenter study conducted in Argentina on a larger number of cases of BD (*n* = 714) found a Stx positivity rate of 4.1% [8].

**Table 1:** Distribution of pathogens identified in children with acute bloody diarrhea by molecular microbiology (*n* = 1727)

Pathogen	Bloody diarrhea (%)
*Campylobacter*	37.6
Non-STEC	23.2
*Salmonella* spp.	18.6
STEC	7.1
*Aeromonas* spp.	4.9
*C. difficile*	3.5
*Yersinia* spp.	2.9
EIEC/*Shigella* spp.	2.0
*Vibrio* spp.	0.1

When STEC infection is confirmed, the child faces a 10–20% risk of developing HUS [1, 9]. This risk varies depending on the genetic profile of the STEC strain, as Stx1 is rarely associated with HUS, while Stx2 is linked to HUS at variable but high frequencies [4]. In our experience, the risk of HUS associated with Stx2 is approximately 23% [1]. Additionally, when STEC produces both Stx1 and Stx2, the risk of progression to HUS is lower (approximately 12%). Stxs bind to enterocytes, cross the brush border, and damage the vascular network of the intestinal mucosa, causing hemorrhagic colitis. Once the Stx reaches the circulation, its pentameric B subunit binds to globotriaosylceramid (Gb3Cer), which is well expressed on the microvascular endothelium. Upon binding to Gb3Cer, Stxs are internalized via endocytosis and transported through the retrograde pathway to the trans-Golgi network, thus reaching the endoplasmic reticulum (ER). At this stage, the enzymatic A subunit is released into the cytoplasm, interfering with protein synthesis and leading to apoptosis, ER stress, inflammation, and further cellular damage [10].

Therefore, in a child with BD, diagnostic tests for the presence of Stx are essential and should be performed as soon as possible. In summary, out of 100 children who present with BD, at least 5–7 will test positive for Stx, and one will ultimately develop HUS. Finally, while STEC-HUS can certainly affect adults as well, the epidemiological scope of the problem beyond the pediatric age is largely unknown.

## Diagnostic tests for Stx

In the past, traditional microbiological methods available in diagnostic laboratories could only detect STEC strain O157 through culture. This may explain why O157 has long been considered the primary pathogen responsible for HUS. However, more recent case series have shown that this is no longer the prevalent strain in many countries, including European countries [11, 12].

With the advent and spreading of molecular tests, serotyping is no longer required in the initial diagnostic phase. When confronted with a case of BD, the critical test is the detection of the Stx genes. This test should be performed in all children presenting with BD. If the result is negative, the test may be repeated to avoid false negative results.

Identifying the specific serotype responsible for the infection remains useful for epidemiological investigation, but for diagnostic purposes, it is sufficient to determine whether the Stx2 gene is expressed, as its expression is associated with a well-defined risk of HUS, as previously mentioned.

Currently, several genomic tests, all of which are highly sensitive, although sometimes less specific, are commercially available for Stx gene identification. Owing to their high sensitivity, it is not uncommon to detect multiple positive results. In such cases, it is advisable to consider the infection as potentially linked to the disease that is more severe and act accordingly.

Multiplex PCR analysis, which uses primers and probes targeting specific pathogen sequences, allows simultaneous identification of several pathogens, including STEC, with a single analysis. Subsequent bacterial isolation and detection of specific antibodies (anti-lipopolysaccharide) can provide a more accurate and comprehensive diagnosis.

Other diagnostic strategies are also available for confirming or excluding a STEC infection in patients with BD. Among these are rapid tests for the identification of Stx in stool samples (EIA) or for the detection of specific IgM antibodies by Glyco-iELISA. These tests, while having the advantage of providing a rapid bedside diagnosis of STEC infection, are limited by lower sensitivity (EIA) or by the ability to detect only a few serotypes (for the second method). As a result, they are most useful in specific epidemiological contexts or if positive because a negative result does not rule out STEC infection and the consequent risk of HUS (low sensitivity).

## Management of STEC infection

**Fig. 1:**
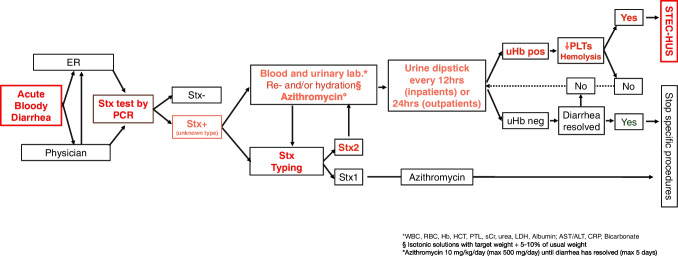
Management of bloody diarrhea focused on the risk of STEC-HUS in children at our Center. FER, emergency room; Stx, shiga toxin; PCR, polymerase chain reaction; uHb, urinary hemoglobin; WBC, white blood cells; RBC, red blood cells; Hb, hemoglobin; HCT, hematocrit; PTL, platelet count; sCr, serum creatinine; LDH, lactate dehydrogenase; AST, aspartate aminotransferase; ALT, alanine aminotransferase; CRP, C-reactive protein; STEC-HUS, hemolytic uremic syndrome associated with Shiga toxin-producing *Escherichia coli* infection

We suggest monitoring patients with suspected or confirmed STEC infection for the possible onset of HUS through regular urine dipstick tests for the detection of hemoglobinuria every 12 h (in hospitalized patients) or every 24 h (in patients not requiring hospitalization) until diarrhea has resolved (Fig. 1) [13]. If only trace amounts of hemoglobin are detected, the test should be repeated shortly after. In case of confirmed hemoglobinuria, blood tests should be performed to rule in or out the diagnosis of ongoing HUS, based on the classic diagnostic triad: 1. platelet consumption (platelets count < 150,000/mm^3^ or its acute reduction); 2. hemolysis (hemolytic anemia or elevated lactic dehydrogenase or undetectable haptoglobin); and 3. kidney damage (elevated serum creatinine or microhematuria with proteinuria). If the triad is present, the patient has developed HUS and should be referred to a tertiary care center equipped to perform dialysis, if necessary, in a timely manner, as approximately 50% of affected patients may require acute kidney replacement therapy [14].

**Fig. 2:**
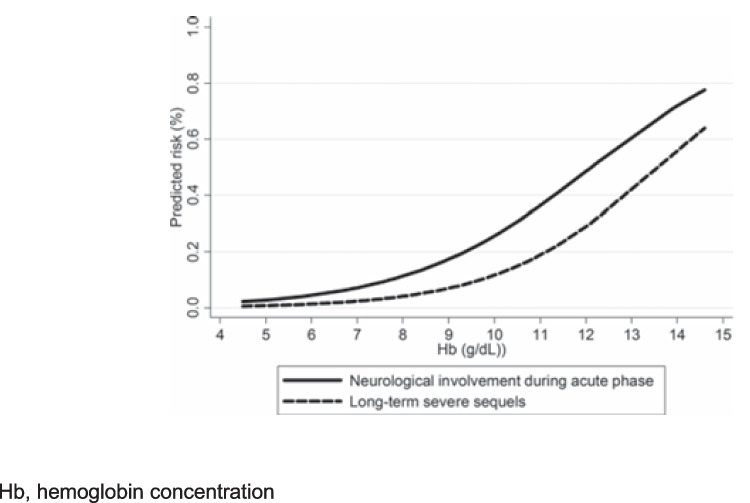
Relationship between hemoglobin concentration at STEC-HUS onset and disease severity. Hb, hemoglobin concentration

**Fig. 3:**
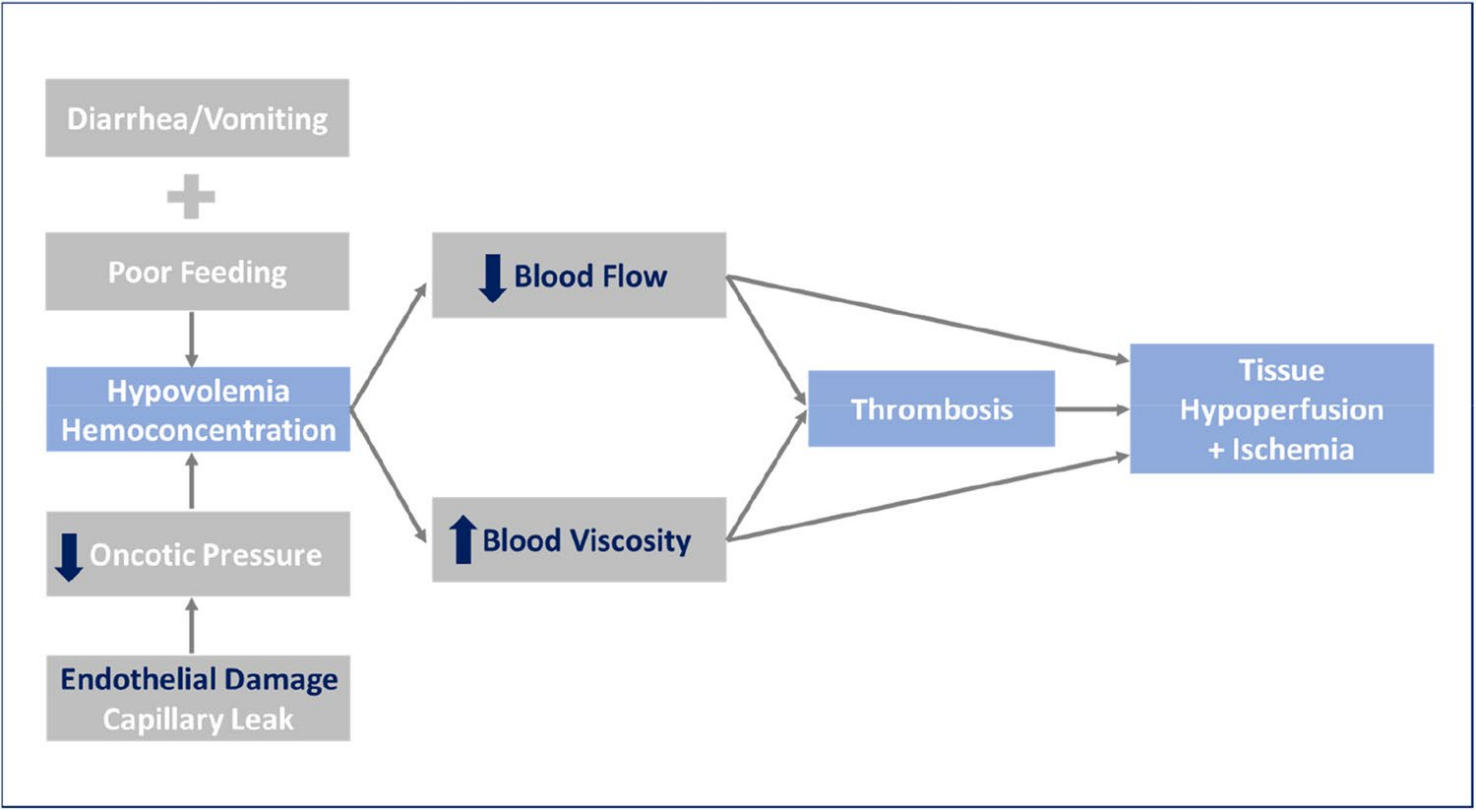
Pathophysiology of the general relationship between hemoconcentration and disease severity in thrombotic microangiopathies

Pending diagnostic results patients should be managed based on their hydration status, overall clinical conditions, ability to tolerate oral fluids, and family compliance. Whenever feasible, if Stx2 is detected (alone or together with Stx1), hospitalization is advisable to allow for close monitoring and appropriate fluid management. Alternatively, if the patient is clinically stable, well-hydrated, tolerating oral fluids, and the family is reliable, outpatient management with continued oral rehydration and regular urine dipstick monitoring may be appropriate. Intravenous rehydration and volume expansion are critical to provide optimal nephroprotection, as it is well established that, in case of STEC-HUS development in a patient with intravascular dehydration, the subsequent disease course and complications are likely to be more severe (Fig. 2) [15–17]. Early generous fluid infusion can reduce microvascular thrombi formation and the consequent ischemic organ damage due to hypoperfusion (Fig. 3), with positive effects on both short- and long-term disease outcomes. Adequate volume expansion should be assessed by echocardiography with measurement of cardiac output or vena cava collapsibility, particularly if oligo-anuria coexist, to prevent volume overload [17, 18]. Intravenous rehydration and/or volume expansion should be obtained using an isotonic crystalloid solution with a high sodium chloride concentration (130–154 mEq/L), or a balanced salt solution (e.g., lactated Ringer’s). The latter is preferable when prolonged infusion is needed. It is crucial to use an isotonic solution to prevent acute hyponatremia due to the dilution of plasma sodium in the case of sudden oligo-anuria associated with HUS onset.

Antibiotic use in STEC-infected patients has historically been contraindicated [19, 20]. Antibiotic treatment seems to increase the risk of STEC-HUS development by inducing prophages containing Stx-encoding genes through the bacterial SOS response, thereby promoting Stx production, switching from a lysogenic state to a lytic cycle and Stx release from dead bacteria via phage-mediated lysis [21, 22]. By favoring the release of Stx-encoding phages, it may also precipitate the infection of nonpathogenic intestinal *E. coli* and facilitate further attachment of STEC to the gut wall by eliminating competing commensal intestinal flora [23, 24].

However, current recommendations are primarily based on studies that do not distinguish between different classes of antibiotics, combining bacteriostatic and bactericidal drugs. Several in vitro studies have demonstrated that the effect of antibiotics on toxin release depends on the type and concentration of antibiotics used and differs among STEC strains [23, 25]. Moreover, during the 1996 outbreak in Japan, patients infected with STEC were extensively treated with antibiotics (especially fosfomycin) as the Japanese Ministry of Health and Welfare advocated their use [26]. Japanese researchers reported favorable outcomes with this approach [27, 28], although the supporting evidence remains relatively weak.

Indeed, the available literature shows that bactericidal antibiotics, such as quinolones, beta-lactams, and cephalosporins, increase Stx production in vitro and therefore may increase the risk of progression to HUS [29, 30]. In contrast, emerging evidence supports the benefits of the use of bacteriostatic agents, such as transcriptional and translational inhibitors. Azithromycin has been shown to reduce Stx production in vitro [30–33] and to exert protective effects against neurological and gastrointestinal complications associated with STEC infections in animal models [34]. Data extrapolated from the 2011 STEC O104:H4-HUS outbreak in Germany not only revealed no evidence that azithromycin worsened the clinical course in patients with severe STEC-HUS who received prophylaxis for meningitis before being administered eculizumab but also indicated that it shortened the duration of bacterial shedding in long-term carriers [35].

Moreover, a recent retrospective study evaluating the effect of the use of different antimicrobial classes during the first week of STEC-related diarrhea showed that none of the patients treated with azithromycin developed HUS, although the sample was small, it combined children (at higher risk) with adults, and the protective association did not reach statistical significance [36]. However, earlier studies in small case series (*n* = 5) failed to demonstrate a protective effect of azithromycin treatment [9, 37].

In Denmark, azithromycin is currently recommended for STEC infection under specific conditions, primarily for decolonization purposes [38, 39], while the use of azithromycin in patients affected by STEC-HUS is currently being assessed in a single-center clinical trial (NCT02336516). Moreover, in a recent retrospective analysis conducted at our center that included 57 patients treated with azithromycin (10 mg/kg/day for 5 days), only one patient developed HUS, whereas the expected number was eight. This corresponds to a conversion rate of 1.8%, compared to a historical rate of 15.0% [40]. At our center, all isolated STEC from humans are tested for antibiotic resistance, and so far out of 127 tested, none was resistant to azithromycin. However, it is obvious that an increased use of this bacteriostatic agent in the setting of STEC infection might favor the development of resistance [41].

Nevertheless, the ability of azithromycin to prevent HUS has not been proven yet, thus the administration of antibiotics in the diarrheal phase of STEC infections should only be considered in the context of a controlled clinical trial, when there are sufficient data in support of their ability to prevent kidney injury in these patients.

## STEC-HUS management

Even when HUS is already ongoing, patients may still benefit from early volume expansion (as soon as possible), with rapid isotonic fluid infusion (10 mL/kg/h), which has already been shown to reduce the disease severity [15–17, 42–44]. Nevertheless, a multicenter crossover randomized clinical trial (NCT05219110) is currently ongoing to confirm whether early high-volume intravenous fluid administration can effectively improve kidney outcomes in STEC-infected children. Once established, HUS follows a highly variable course. Factors such as the presence of anuria, a white blood cell count > 20,000/mm^3^, and elevated Hb at onset are all predictive of more severe disease and worse outcomes [2, 3, 14].

In addition to the already mentioned procedures, the management of STEC-HUS is based on supportive care, but the systematic analysis of such measures goes beyond the scope of this paper, which focuses on the early stages of the disease.

In STEC-HUS red blood cells transfusions are rarely avoidable. To minimize mechanical hemolysis and avoid an excessive increase of free Hb levels, the transfusion regimen and target should be tailored primarily to the clinical context.

Platelet transfusions should be avoided unless symptomatic. Loop diuretics may be useful in oliguric patients with severe fluid overload but are rarely helpful in anuric subjects. Diuretics regain their potential therapeutic role when endothelium repairs, capillary leak ceases, and plasma albumin level normalizes, leading to fluid recall from the third space, and a transient state of hypervolemia and hypertension. Indeed, high blood pressure is uncommon at disease onset because of the already mentioned hypovolemia (diarrhea, vomiting, poor feeding, fever, and capillary leak), while it plays a role during the recovery phase.

Despite the analogy with atypical HUS and the amount of data produced to support the involvement of the complement system and therefore the use of C5 inhibitors in STEC-HUS, a recent French randomized controlled trial documented that these drugs have little or no space at all in this clinical setting [45]. Obviously, cases of BD repeatedly negative to Stx may hide the onset of aHUS and must be further investigated to rule in or out complement dysregulation. A phase 3 study is currently ongoing on the use of equine polyclonal antibodies against Stx for STEC-HUS (NCT06389474).

## Outcome of STEC-HUS

Approximately 50% of patients with STEC-HUS will require dialysis (either extracorporeal or peritoneal, depending on the experience of the center with these procedures). Between 10 and 20% of patients develop neurological symptoms, primarily seizures. Central nervous system involvement remains the leading cause of death, and the case fatality rate is 1–3%. After a variable period (between 5 and 10 days), thrombotic microangiopathy (TMA) typically resolves spontaneously with a normalization of the platelet count and, hopefully, an improvement in kidney function. Anemia continues for a few more days because of the persistent destruction of erythrocytes caused by the residual thrombi, until they are dissolved. Complete kidney recovery may take a long time, sometimes years [46]. Approximately 50% of patients fully recover, although the loss of nephrons may lead to long-term consequences (preeclampsia, hypertension, and CKD), even decades later. The remaining patients may suffer from varying degrees of kidney damage, mainly proteinuria and chronic kidney insufficiency. Fewer than 5% of patients will remain dependent on dialysis or have permanent neurological damage [14].

## Conclusions

The widespread use of molecular microbiology has made it possible to diagnose STEC infection before HUS onset. Children with acute BD should be screened for STEC infection and positive patients should be treated with fluid administration and carefully monitored for the possible development of the TMA.

## Key summary points

1. Acute BD in children should always be considered a potential clinical emergency.
2. Depending on the season, 5–20% of children with acute BD are infected with STEC. Among STEC-infected patients, 10–15% develop HUS, especially if Stx2-positive.
3. The standard diagnostic test for STEC infection is the detection of Stx using molecular microbiology. In children with BD, diagnostic testing for the presence of Stx is essential.
4. Once STEC infection is confirmed, patients should undergo early and generous fluid administration to induce a mild volume expansion.
5. Stx-positive patients should be monitored for HUS onset using urine dipstick testing for hemoglobinuria.
6. Bacteriostatic antibiotics have shown promising results in the secondary prevention of STEC infection. However, their use should only be considered in the context of a controlled clinical trial.

## Multiple choice questions

Answers appear following References.

1. Which of the following test is more useful for identifying early-onset HUS during acute BD?
  a Serum creatinine
  b Haptoglobin
  c Urine dipstick for hemoglobinuria
  d Blood count
2. Which of the following measures is not part of the supportive therapies used in STEC-HUS?
  a Intravenous hydration.
  b Transfusion of packed red blood cells.
  c Hemodialysis.
  d Platelet transfusion.
3. What is the best way to diagnose STEC infection?
  a Rapid tests for Stx in stool samples (EIA).
  b Stool culture.
  c Glyco-iELISA for specific IgM antibodies.
  d Molecular detection of Stx genes.

## Supplementary Information

Below is the link to the electronic supplementary material.

Graphical abstract (PPTX 607 KB)
