# Supplementary material for: Bloody diarrhea, STEC infection, and HUS in the molecular microbiology era
Source: Pediatr Nephrol. 2025 Aug 23;41(4):973–80. doi: 10.1007/s00467-025-06930-y (PMC12953460; doi:10.1007/s00467-025-06930-y)
Supplement: Supplementary file 1 — Graphical abstract (PPTX 607 KB) [file 467_2025_6930_MOESM1_ESM.pptx]

## Slide 1
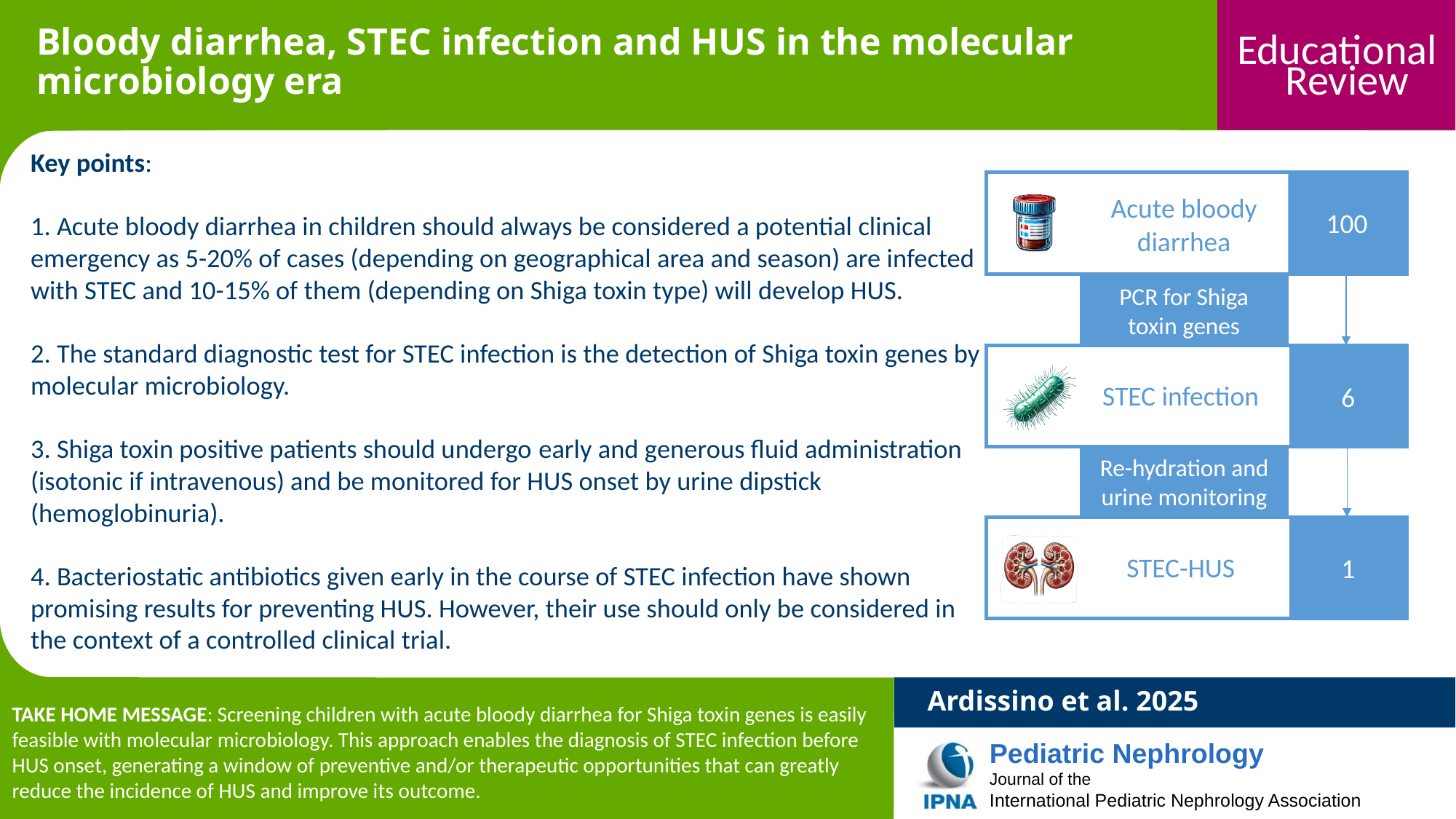

Bloody diarrhea, STEC infection and HUS in the molecular microbiology era
Key points:
1. Acute bloody diarrhea in children should always be considered a potential clinical emergency as 5-20% of cases (depending on geographical area and season) are infected with STEC and 10-15% of them (depending on Shiga toxin type) will develop HUS.
2. The standard diagnostic test for STEC infection is the detection of Shiga toxin genes by molecular microbiology.
3. Shiga toxin positive patients should undergo early and generous fluid administration (isotonic if intravenous) and be monitored for HUS onset by urine dipstick (hemoglobinuria).
4. Bacteriostatic antibiotics given early in the course of STEC infection have shown promising results for preventing HUS. However, their use should only be considered in the context of a controlled clinical trial.
100
Acute bloody diarrhea
PCR for Shiga
toxin genes
6
STEC infection
Re-hydration and
urine monitoring
1
STEC-HUS
Ardissino et al. 2025
TAKE HOME MESSAGE: Screening children with acute bloody diarrhea for Shiga toxin genes is easily feasible with molecular microbiology. This approach enables the diagnosis of STEC infection before HUS onset, generating a window of preventive and/or therapeutic opportunities that can greatly reduce the incidence of HUS and improve its outcome.
